# Expert Consensus on SABA Use for Asthma Clinical Decision-Making: A Delphi Approach

**DOI:** 10.1007/s11882-023-01111-z

**Published:** 2023-11-22

**Authors:** Njira Lugogo, Maeve O’Connor, Maureen George, Rajan Merchant, Greg Bensch, Jay Portnoy, John Oppenheimer, Mario Castro

**Affiliations:** 1 Division of Pulmonary & Critical Care Medicine, Department of Internal Medicine, University of Michigan, Ann Arbor, MI USA; 2 Allergy Asthma and Immunology Relief, Charlotte, NC USA; 3 Columbia University School of Nursing, New York, NY USA; 4 Woodland Clinic Medical Group, Allergy Department, Dignity Health, Woodland, CA USA; 5 Allergy Immunology and Asthma Medical Group, Stockton, CA USA; 6 Section of Allergy, Asthma & Immunology, Children’s Mercy Hospital, University of Missouri-Kansas City School of Medicine, Kansas City, MO USA; 7 Department of Internal Medicine, New Jersey Medical School, Newark, NJ USA; 8 Pulmonary and Allergy Associates, Morristown, NJ USA; 9 Division of Pulmonary, Critical Care, and Sleep Medicine, Department of Internal Medicine, University of Kansas Medical Center, Kansas City, KS USA

**Keywords:** Asthma, Reliever inhaler, Delphi consensus, SABA overuse, Asthma guidelines, Exacerbations

## Abstract

**Purpose of Review:**

A modified Delphi process was undertaken to provide a US expert-led consensus to guide clinical action on short-acting beta_2_-agonist (SABA) use. This comprised an online survey (Phase 1), forum discussion and statement development (Phase 2), and statement adjudication (Phase 3).

**Recent Findings:**

In Phase 1 (*n* = 100 clinicians), 12% routinely provided patients with ≥4 SABA prescriptions/year, 73% solicited SABA use frequency at every patient visit, and 21% did not consult asthma guidelines/expert reports. Phase 3 experts (*n* = 8) reached consensus (median Likert score, interquartile range) that use of ≥3 SABA canisters/year is associated with increased risk of exacerbation and asthma-related death (5, 4.75–5); SABA use history should be solicited at every patient visit (5, 4.75–5); usage patterns over time, not absolute thresholds, should guide response to SABA overuse (5, 4.5–5).

**Summary:**

Future asthma guidelines should include clear recommendations regarding SABA usage, using expert-led thresholds for action.

**Supplementary Information:**

The online version contains supplementary material available at 10.1007/s11882-023-01111-z.

## Introduction

Asthma affects 25.3 million people in the USA, and 60% of adults and 44% of children have uncontrolled asthma as defined by the National Asthma Education and Prevention Program (NAEPP) [1].

Overuse of short-acting beta_2_-agonists (SABAs) for as-needed symptom relief has repeatedly been found to be common in uncontrolled asthma [2–4, 5••, 6, 7, 8••, 9], though the definition of “overuse” varies across studies [2, 4]. High SABA use is associated with exacerbations, asthma-related hospitalizations, and increased risk of asthma-related death [5••, 6, 10••, 11–13]. Progressively increasing SABA use has been observed from 10 to 14 days before an exacerbation [14]; thus, identifying and acting upon such periods may halt exacerbation progression and improve outcomes [6, 15].

Understanding and monitoring SABA use is a cornerstone of asthma management; however, managing SABA use is challenging [11, 16–21]. Although asthma guidelines acknowledge the problem of SABA overuse, define it in broad terms, and advise intervention if it occurs, patients and healthcare systems would stand to benefit from more detailed recommendations for monitoring and next steps. Established methods of monitoring SABA overuse (patient recall and/or prescription refill data) are often subjective or inaccurate [22•]. Furthermore, SABA overprescription, defined as >2 canisters/year [6] or ≥3 canisters/year [8••, 23, 24•], remains prevalent across all asthma severities, and asthma guidelines offer differing advice for SABA prescribing [11, 18–21]. Reliever medications, including SABA, are also available without a prescription in several countries [3, 10••]—potentially including the USA in the future [25]—hindering effective monitoring of overuse. To address these issues, critical exploration of SABA use in asthma is needed.

The modified Delphi method is a well-established approach to answer a research question through expert input to identify a consensus view [26]. Panels typically have 7–15 members to balance diverse representation with the opportunity for intimate group discussion [26]. The method is a systematic and iterative process allowing for reflection, consideration of nuance, and reconsideration of personal opinions in response to those of other experts [27]. This approach is used when current knowledge is incomplete or potentially subjective or when obtaining further evidence is not feasible [28, 29].

The aim of this study was to provide expert-led guidance on appropriate clinical action in response to high SABA use and/or changes in patients’ SABA usage patterns, using a modified Delphi mixed-method process to reach a consensus among a panel of experts in respiratory medicine and asthma management.

## Methods

### Study Design

In 2021, a multiphase, iterative, modified Delphi mixed-method consensus-building process was conducted. The Delphi method has limitations such as lack of standardized consensus-defining methods, problems associated with anonymity, and potential lack of generalizability beyond the experts included [30]. It has therefore been adapted in several ways to fit specific research needs. This study used a mixed-method approach, including an iterative set of interactions with clinicians treating asthma, to gain insight into SABA reliever medication use among patients with asthma.

The project team met twice weekly for approximately 3 months to plan the strategic approach to this research. As part of this planning phase, a targeted review of existing literature on SABA reliever medication use was conducted. Searches in PubMed and Google Scholar, conducted in September 2021, included combinations of the following search terms: asthma, short-acting beta antagonist, SABA, rescue inhaler, rescue medication, albuterol, use, overuse, abuse, burst, and treatment patterns. Through team discussion and input from the panel Chair, key themes were extracted from the literature findings and used to inform the development of study domains. These would serve as a framework for ensuring all aspects of SABA use were addressed in the subsequent study design and development of study-related materials. As a result of the extensive literature search, a total of 14 SABA use domains were identified: overuse (volume/amount/quantity/frequency), appropriate use, reducing risk, exacerbations, practice guideline alignment, objective testing (of disease severity), socioeconomics, efficacy, safety, disease severity/burden, relationship between maintenance and reliever therapy, SABA canister dispensing control and monitoring to prevent overuse, healthcare resource utilization (HRU), and shared decision-making.

**Fig. 1:**
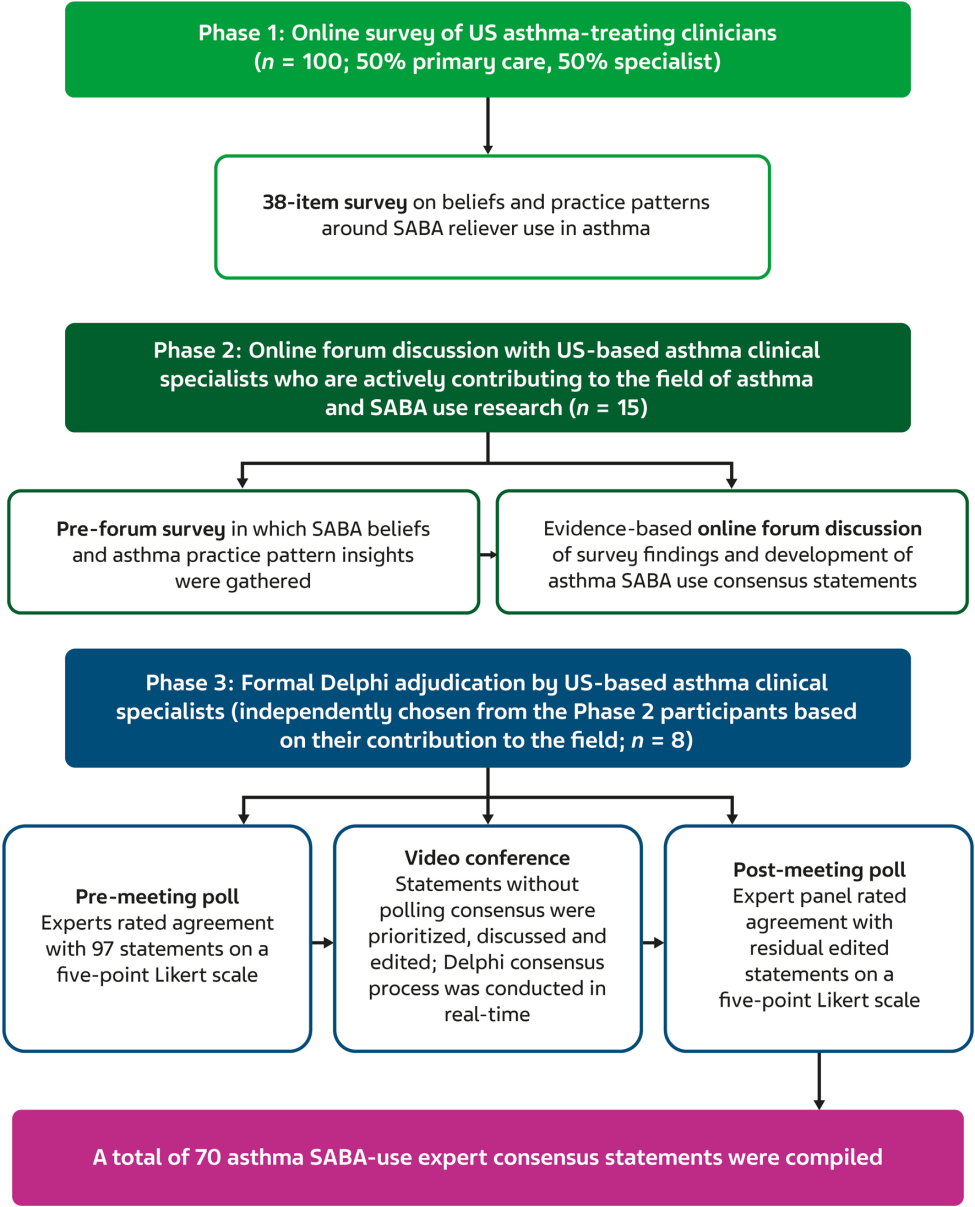
Study design. *Experts rated statements on a five-point Likert scale, ranging from (1) strongly disagree to (5) strongly agree. SABA, short-acting beta_2_-agonist

The three-phase study comprised (1) an anonymous online survey exploring SABA asthma reliever beliefs and real-world practice, (2) an anonymous forum discussion with comprehensive evidence review and SABA statement development, and (3) an expert-guided formal modified Delphi adjudication of generated statements (Fig. 1; Appendix 1).

Key study objectives were to gather clinician insights on asthma reliever medication use in real-world practice, gain an understanding of practice-based clinical decision-making related to asthma reliever medications (including SABA), identify educational needs to support healthcare professional’s (HCP) confidence in using objective inhaler use data for clinical management, and develop consensus statements.

### Participants

Eligible Phase 1 participants, recruited through a third-party clinician panel, had 2 to 30 years of clinical experience, were currently treating patients with asthma in the USA (excluding Vermont and Maine as HCPs cannot participate in paid forums), and were primary care physicians (PCPs) or specialists (allergists, pulmonologists). Phase 2 panelists were nationally known asthma clinical specialists (identified by Sensified, LLC through a search of professional societies, published literature, and treatment guidelines) currently managing patients with asthma in the US and actively researching asthma and SABA use. Phase 3 panelists were a subset of Phase 2 participants and were independently chosen by Sensified, LLC to continue to Phase 3 based on their contribution to the field.

## Results

### Phase 1

One hundred clinicians from 48 states plus Washington, D.C., completed the survey. These clinicians comprised 50 PCPs (38 family medicine, 10 adult primary care, 2 pediatric primary care) and 50 specialists (35 allergists, 15 pulmonologists), averaging 21.3 years in practice. Mean number of patients seen was 63/week. Full survey results are listed in Appendix 5.

For SABA prescriptions and refills, 76% PCPs provided 1–3 SABA refills to patients per prescription versus 66% of allergists and 100% of pulmonologists, and 20% of PCPs and 6% of allergists provided ≥4 SABA refills per prescription. Overall, 21% of Phase 1 study participants reported that their practice provided >1 SABA canister/prescription over half of the time.

Several questions addressed SABA use frequency and asthma control. When asked the lowest number of SABA episodes/week likely to be representing an impending or ongoing asthma exacerbation, 24% (PCPs), 34% (allergists), and 13% (pulmonologists) said ≥3 SABA episodes/week. Additionally, 18%, 31%, and 33%, respectively, deemed that ≥5 SABA episodes/week likely represent an impending or ongoing asthma exacerbation. Reasons for exceeding a SABA use level that clinicians considered appropriate could represent impending or ongoing exacerbations (80% of clinicians); a loss of asthma control (79%); inappropriate use (66%); an impending urgent, emergent, or hospital visit for asthma (63%); or inhaler technique challenges (61%). When clinicians were asked to specify which of these scenarios were most often represented in their practice when a patient exceeded the appropriate level of SABA use, the responses were a loss of asthma control (53% of clinicians), an impending or ongoing exacerbation (29%), an impending urgent, emergent, or hospital visit for asthma (11%), inappropriate use (6%), and inhaler technique challenges (1%).

Regarding how often clinicians seek information on SABA use history: 73% of overall participants (56% PCPs, 91% allergists, 87% pulmonologists) indicated at every visit; 22% (34% PCPs, 9% allergists, 13% pulmonologists) indicated at most visits; and 5% (10% of PCPs only) indicated occasionally, depending on factors for the visit.

Clinicians were asked what clinical actions they implement when SABA overuse was identified. Most participants (76%) overall thought that a medication change should be considered. Other responses included inhaler technique training or additional information gathering (60% of clinicians), an asthma education refresher (56%), or a specialty referral (22%).

**Fig. 2:**
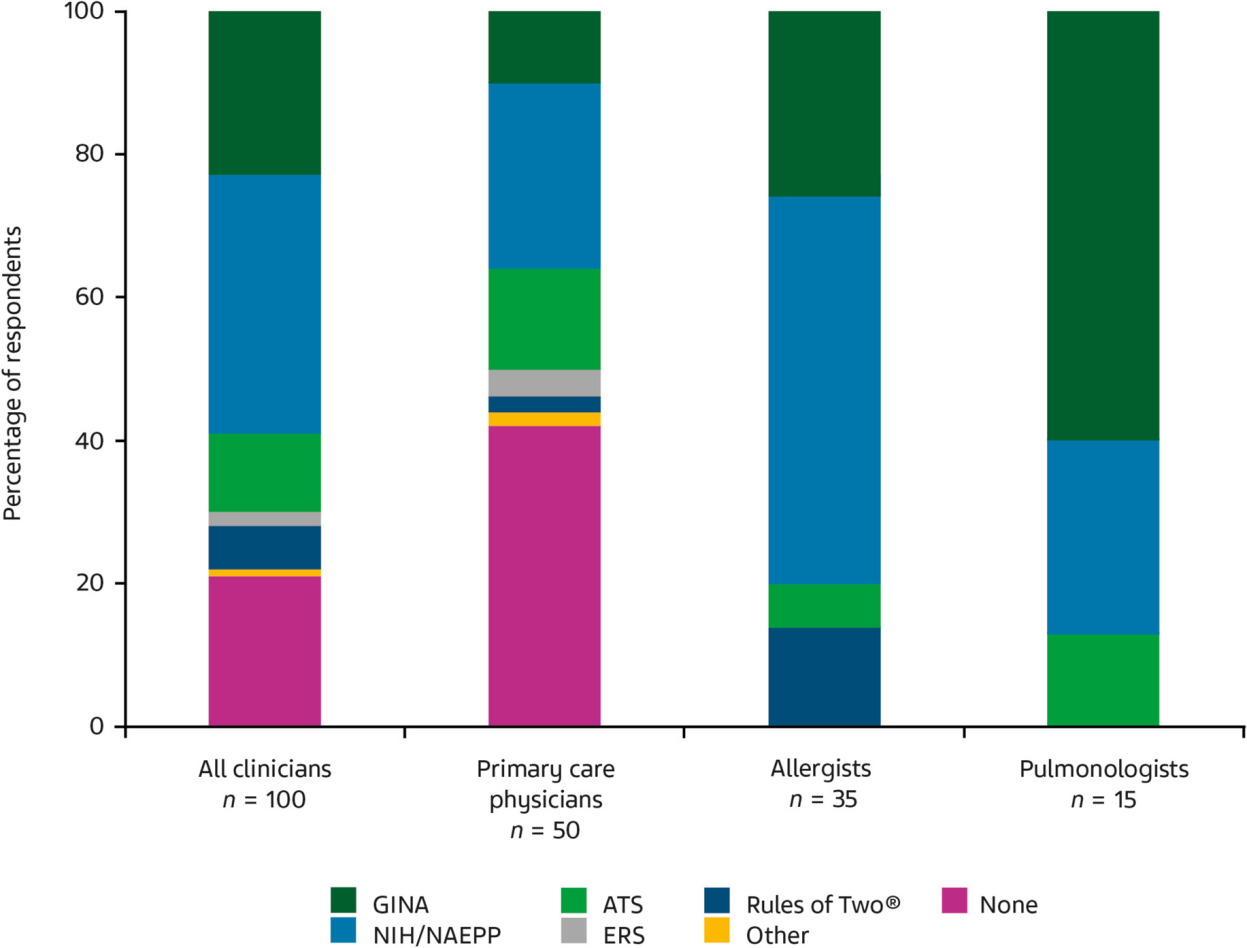
Responses to the Phase 1 survey question “*In your asthma management practice, which (if any) of the following asthma guideline/expert report recommendations do you routinely use for SABA rescue medication guidance*?” 100 US clinicians completed the Phase 1 survey. Participants were required to select the option they most frequently used. ATS, American Thoracic Society; ERS, European Respiratory Society; GINA, Global Initiative for Asthma; NIH/NAEPP, National Institutes of Health/National Asthma Education and Prevention Program

Regarding use of asthma control evaluations, 40% of all clinicians (56% PCPs, 17% allergists, 40% pulmonologists) did not use any validated asthma control survey (e.g., Asthma Control Test [31], Asthma Control Questionnaire) for assessing patients with asthma. Figure 2 shows which asthma guidelines and expert reports were used for SABA use guidance. The National Institutes of Health (NIH/NAEPP) guideline was used by 36% of clinicians (26% PCPs, 54% allergists, 27% pulmonologists). Overall, 23% of clinicians (10% PCPs, 26% allergists, 60% pulmonologists) followed Global Initiative for Asthma (GINA) recommendations. Other guidelines included European Respiratory Society (2% of all clinicians), American Thoracic Society (11%), and Baylor University Rules of Two^®^ (6%). However, 21% of clinicians (42% PCPs) did not use any SABA medication guidance.

Of all Phase 1 study participants, 80% agreed or strongly agreed that the asthma guidelines or expert reports they use provide clear recommendations regarding effective and safe amounts of SABA reliever. Moreover, guidelines were perceived to provide clear recommendations on when to take clinical action for SABA overuse (81% agreed or strongly agreed) and what specific action to take (83% agreed or strongly agreed). However, experts in Phase 3 (see below) did not agree with these perceptions.

### Phase 2

The pre-meeting survey (Appendix 2) was completed by 10/15 expert panelists. Most panelists (80%) deemed exceeding an appropriate SABA use to be associated with high or very high risk for negative outcomes. At least 90% said healthcare costs, HRU, and quality of life would be improved if exacerbations associated with SABA use could be lessened in severity or prevented. In contrast to the Phase 1 study participants, 60% of expert panelists said that literature, expert reports, and/or guidelines did not provide clear recommendations for effective and safe thresholds of SABA medication use. Furthermore, 70% of expert panelists noted that no clear recommendations were provided on what clinical action to take, and when, in response to high SABA use.

All 15 panelists participated in the online forum discussion. Panelists provided thoughts on five patient cases and their likely response. Panelist responses largely referenced concern about potential exacerbations or loss of disease control and a desire for more patient-specific information. The most common clinical actions that might be taken by the group for a patient reaching the threshold for appropriate SABA use included: adjustment of therapy (increase or addition of therapies [73% of panelists], oral corticosteroid [40%]), consideration of triggers (53%), further evaluation (47%), and adherence assessment (40%).

### Phase 3

Overall, 97 statements were compiled from Phases 1 and 2 encompassing nine topics: gathering information on disease and SABA use, patient SABA use history, SABA prescribing, SABA use levels, exacerbations, disease control, clinical actions and SABA use levels, socioeconomics and SABA use, and guidelines. In the pre-meeting poll, eight participants (five allergists, two pulmonologists, one nurse scientist) scored these statements; 61 were accepted, 6 were rejected, and 30 required revisions that were discussed in the video conference. Through the video conference and post-meeting poll, 23 modified statements were scored and of these 9 were accepted and 14 rejected. Thus, the expert panel consensus consisted of 70 accepted statements relating to eight topics (Table 1); the topic “guidelines” did not gain consensus.

**Table 1:** All consensus statements agreed upon during Phase 3

**Gathering information on disease and SABA reliever medication use**	**Median Likert score (IQR)**
1. The ACT or ACQ or another validated asthma control survey should be used as part of routine assessment of patients with asthma.	5 (5–5)
2. The amount of a patient’s rescue medication use should be part of their asthma medical history evaluations.	5 (5–5)
3. For patients who have been prescribed SABA rescue medication (e.g., albuterol) for asthma, information about previous rescue medication use in the prior weeks or months should be obtained at every visit.	5 (5–5)
4. Patient history should be used to assess SABA rescue medication use for patients with asthma.	4.5 (4–5)
5. Clinicians should solicit information on frequency of SABA use at every encounter with an asthma patient.	5 (4.75–5)
6. Pharmacy refill data could be used to assess SABA reliever medication use for patients with asthma; however, these data may not correlate with actual SABA reliever use.	4 (4–4.25)
7. Digital health tools should be used to assess SABA rescue medication use for patients with asthma.	4 (4–5)
8. A patient’s asthma rescue medication use should factor into clinical decision-making for asthma management.	5 (4–5)
9. A better understanding of SABA rescue medication overuse should play a role in shared decision-making between patients with asthma and healthcare professionals in the United States.	4 (4–5)
**Patient SABA use history**	
10. Patient SABA use history is generally accurate, but other information (e.g., validated questionnaires, refill data, digital recorders) should be used to obtain accurate information on the patient’s SABA use since their previous visit.	4 (4–4.25)
11. The accuracy of patient SABA use history is variable, and its use should depend on the patient.	4 (4–4)
12. The accuracy of patient SABA use history may be inaccurate and therefore should not be used as the sole determinant of the patient’s SABA use.	5 (5–5)
13. SABA reliever use frequency as gathered in patient history plays a role in the assessment of a patient’s asthma control.	4 (4–4.25)
14. SABA reliever use frequency plays a role in the assessment of a patient’s asthma control. The reliability of the information utilized should be taken into consideration.	4.5 (4–5)
**SABA reliever medication prescribing**	
15. Patients should have SABA refills available, but refill rates should be monitored closely. Use of 3 or more canisters a year is associated with an increased risk of exacerbations and asthma related death.	5 (4.75–5)
**Levels of SABA reliever medication use**	
If a patient exceeds the level of SABA rescue medication use you feel is appropriate, they may be at risk of	
16. Loss of asthma control	4.5 (4–5)
17. An impending or experiencing an ongoing asthma exacerbation	4 (4–5)
18. An impending, urgent, emergent or hospital visit for asthma	4 (4–5)
19. Inappropriate SABA use	4 (4–4)
20. Experiencing inhaler technique challenges	4 (4–4.25)
If a patient exceeds the level of SABA rescue medication use you feel is appropriate, they are likely to be at risk of	
21. Loss of asthma control	4 (4–5)
22. An impending or experiencing an ongoing asthma exacerbation	4 (4–5)
23. An impending urgent, emergent or hospital visit for asthma	4 (4–5)
**Exacerbations**	
24. The use of SABA reliever medication 2–3 times per week may represent an impending or ongoing asthma exacerbation. The magnitude of an individual’s increase above their baseline in reliever SABA use is important and clinical correlation is essential.	4 (4–5)
The following amount of weekly SABA rescue medication use likely represents an impending or ongoing asthma exacerbation:	
25. 5 or more episodes of SABA rescue medication use per week	4.5 (4–5)
26. 7 or more episodes of SABA rescue medication use per week	5 (4–5)
27. 10 or more episodes of SABA rescue medication use per week	5 (4.75–5)
28. 15 or more episodes of SABA rescue medication use per week	5 (4.75–5)
29. 20 or more episodes of SABA rescue medication use per week	5 (5–5)
30. 25 or more episodes of SABA rescue medication use per week	5 (5–5)
31. The patient’s baseline SABA rescue medication use should be considered when determining whether the current weekly use may indicate an impending or ongoing exacerbation.	4 (4–4.25)
32. If the patient’s current SABA rescue medication use is 50% higher than their baseline use, this likely represents an impending or ongoing exacerbation.	4 (4–4)
33. If the patient’s current SABA rescue medication use is 100% higher than their baseline use or more, this likely represents an impending or ongoing exacerbation.	5 (4.75–5)
34. The pattern of SABA use over time should play a role in determining whether SABA rescue medication use might represent an exacerbation.	4 (4–5)
35. The patient’s SABA rescue medication use pattern over time is more useful than an average of weekly SABA rescue medication use for determining whether a patient may be experiencing an impending or ongoing exacerbation.	4 (4–4.25)
36. Valid historical data about night-time SABA asthma rescue medication use should factor into a clinician’s level of concern about asthma exacerbations.	4 (4–4.25)
37. If it were possible, knowing of an impending exacerbation days in advance would allow for an outpatient medication intervention that could prevent the exacerbation.	4.5 (4–5)
**Disease control**	
38. The patient’s baseline SABA rescue medication use should be considered when determining whether the current weekly use may indicate a loss of asthma control.	4 (4–4.25)
39. The pattern of SABA use over time should play a role in determining whether SABA rescue medication use might represent a loss of asthma control.	4 (4–4)
40. The patient’s SABA rescue medication use pattern over time is more useful than an average of weekly SABA rescue medication use for determining loss of asthma control.	4 (4–4)
41. SABA rescue medication overuse could indicate suboptimal effectiveness of a patient’s asthma maintenance therapy.	4 (4–4)
There is a correlation between overuse of SABA rescue medications and	
42. ER visits	4 (4–4)
43. Hospitalizations	4 (4–4)
44. Unscheduled office/practice visits	4 (4–4.25)
45. Increased healthcare costs	4 (4–4)
46. Missed work/school	4 (4–4)
**Clinical actions and SABA reliever medication use levels**	
47. Additional information gathering via phone/portal should be considered if a patient is overusing their SABA rescue medication.	4 (4–5)
48. A medication change should be considered if a patient is overusing their SABA rescue medication.	4.5 (4–5)
49. An asthma education refresher should be considered if a patient is overusing their SABA rescue medication.	4 (4–5)
50. Inhaler technique training should be considered if a patient is overusing their SABA rescue medication.	5 (5–5)
51. Clinical actions in response to a patient’s use of their SABA reliever medication should not depend on a specific threshold but rather an increase from the patient’s baseline use.	5 (4.5–5)
52. If the current SABA rescue medication use is 100% higher or more than the patient’s baseline, this should warrant additional clinical action.	5 (4.75–5)
The following should influence the decision about how to respond to a patient’s SABA rescue medication overuse:	
53. Severity of symptoms	5 (4.75–5)
54. Disruption of activities	4.5 (4–5)
55. History of exacerbations	5 (4–5)
56. Reports of healthcare utilization (e.g., urgent care, ER, hospitalization)	5 (5–5)
57. History of ICU care	5 (5–5)
58. Level of disease control	5 (4–5)
59. Adherence history	5 (4.75–5)
The following should be considered for patients identified as overusing their SABA rescue medication:	
60. Additional asthma specialty care	4.5 (4–5)
61. Additional inhaler training	4.5 (4–5)
62. Additional asthma education	4.5 (4–5)
An appropriate outpatient asthma therapeutic intervention for a patient with asthma demonstrating excessive SABA rescue medication use could result in	
63. Better disease recognition	4 (4–4.25)
64. Improved asthma education	4 (4–4.25)
65. Enhanced asthma control	4.5 (4–5)
66. Reduction in asthma exacerbation severity	4.5 (4–5)
67. Improved quality of life	4.5 (4–5)
68. Lessened risk of asthma death	4 (4–5)
69. Improved work/school productivity	4 (4–4.25)
**Socioeconomic status and SABA reliever medication**	
70. SES influences a patient’s need for SABA rescue medication use.	4 (4–4)

*ACT* Asthma Control Test, *ACQ* Asthma Control Questionnaire, *ER* emergency room, *ICU* intensive care unit, *IQR* interquartile range, *SABA* short-acting beta_2_-agonist, *SES* socioeconomic status

Several consensus points were noteworthy. Use of ≥3 SABA canisters/year was associated with increased risk of exacerbation and asthma-related death. Patients should have SABA refills, with close monitoring of refill rates. Moreover, ≥5 SABA episodes/week and/or SABA episodes ≥50% and >100% above the patient’s baseline were considered to represent an impending or ongoing asthma exacerbation. History of SABA usage should be solicited at every patient encounter. Reliability of usage frequency should be considered since patient-sourced information may be inaccurate and pharmacy refill data may not correlate with actual use. Consideration should be given to employing digital health tools to assess SABA use. Individual SABA use data (e.g., increase from baseline, usage patterns over time) should guide clinical actions, rather than absolute thresholds.

## Discussion

We undertook a modified Delphi mixed-method consensus-building process, to gain clinician insights on real-world clinical decision-making around SABA use in asthma, with the objective of providing guidance on identification of SABA overuse and appropriate clinical action. US clinicians reported their opinions and experiences concerning real-world SABA use in asthma. Subsequently, a two-step process involving key experts produced 70 consensus statements providing valuable insight into asthma management in the USA relating to SABA use. Importantly, asthma specialists and PCPs participated in this study, thereby ensuring that multiple clinical practice settings treating a variety of patient types were represented.

### The Scale of the Challenge: Defining SABA Overuse and Its Link to Poor Outcomes

It is by now well established that SABA overuse is widespread and is linked to poor asthma outcomes [10••, 12, 32•]. The SABINA study investigators reported that the greater the number of SABA canisters prescribed per year, the greater the odds of patients having uncontrolled asthma [10••].

Several asthma guidelines and expert reports define SABA overuse based on a specified numeric threshold of canisters/year or on rates of usage. GINA state that refill rates of ≥3 SABA canisters/year are associated with an increased risk of severe exacerbations, and rates of ≥12 canisters/year are associated with substantially increased risk of death [11]. They advise that SABA overuse necessitates intervention to improve overall control [11]. Baylor University Rules of Two^®^ guidance states that using SABA for ≥2 days/week or for ≥2 episodes/week signals uncontrolled asthma [16, 19], and NIH/NAEPP guidelines comment that using >1 SABA canister for as-needed symptom relief during 1 month (potentially ≥12 canisters/year) indicates SABA overuse [21].

Phase 1 participants and Phase 3 experts had differing opinions on asthma guidelines’ clarity on SABA use. In Phase 1, the NIH/NAEPP guidelines were the most commonly followed overall and the guidelines of choice for allergists, whereas most pulmonologists preferred GINA recommendations. However, many PCPs surveyed did not consult asthma guidelines or expert reports for SABA guidance. Thus, it is not surprising that adherence to asthma guidelines is often poor, particularly in the primary care setting [33•, 34, 35••].

Consistent with the guidelines, however, our experts agreed that use of ≥3 SABA canisters/year was associated with an increased risk of exacerbations and asthma-related death and recommended that refill rates be monitored closely. In the real world, these thresholds are routinely exceeded. In the multinational SABINA III study, 38% of patients were prescribed ≥3 canisters/year, and some were prescribed ≥13 canisters/year [10••]. To et al. reported that in Canada, 5.3% of patients with asthma (≥65 years) were prescribed ≥6 SABA canisters/year [12]. Worth et al. reported that in Germany, 36 to 38% of patients with asthma were prescribed ≥3 canisters/year, with overuse increasing with increasing asthma severity [32•]. One-fifth of the PCPs in our Phase 1 reported routinely providing ≥4 SABA refills in a year. It was noted in Phase 3 of our study that changing practice around SABA prescribing will be important in addressing the problem of overuse, and a key goal will be the provision of additional guidance around the recommended number of SABA refills.

Since a change to the GINA guidelines in 2019, SABA monotherapy is no longer recommended [11, 36]. Instead, to reduce the risk of serious exacerbation, adults and adolescents with moderate to severe asthma should receive daily inhaled corticosteroid (ICS)-containing treatment. Evidence shows, however, that real-world adherence with ICS is poor [37] and most patients are still receiving SABA monotherapy [38].

### Perception Versus Reality: Self-Reported SABA Use and Asthma Control

The rate of SABA use tends to be underestimated by both patients and physicians, and the ready availability of SABA over the counter as well as the possibility of obtaining prescription refills for up to 12 inhalers at a time likely exacerbates the problem. An Australian study of electronic medical records and questionnaires from 720 people with asthma found potential SABA overuse in >50% of patients, yet only 28% self-reported overuse [39•].

Furthermore, many patients are unaware of the risks of SABA overuse. In a real-world cross-sectional observational study in Australian community pharmacies, surveying 375 patients, 23% of SABA overusers (≥3 occasions per week) considered SABAs to be “safe to use,” compared with 8% of non-high SABA users [40•]. Evidence also suggests that high SABA users are less likely to self-report good or excellent health [40•, 41]. Indeed, it was found that a higher proportion (43%) of SABA overusers experienced side effects of dry mouth, palpitations, tremors, chest tightness, muscle cramps, or headache compared with 31% of non-high SABA users [3].

In addition, evidence suggests that patients overestimate the degree of control of their asthma, and both patients and clinicians have low expectations for effective asthma management [42–44]. Findings from an online survey of ~2500 people in Asia indicated that, of 2198 patients who perceived their asthma to be controlled, 80% had not in fact achieved GINA-defined asthma control. Furthermore, of the 1225 patients with GINA-defined uncontrolled asthma, only 18% correctly perceived that their asthma is not controlled [45]. Similarly, in a cross-sectional observational study of Australian adults, 11.5% of participants had controlled asthma according to GINA guidelines, but a much larger proportion (66.5%) believed their asthma was well controlled [46].

Together, these observations clearly highlight the present unmet need and the importance of addressing SABA overuse and accurately assessing asthma control.

### Getting Personal: Individualizing Asthma Management

Data on SABA use has potential to contribute invaluable insights for risk stratification [47].

GINA indicates that a short-term increase in use of as-needed SABA is associated with increased likelihood of severe exacerbation in the subsequent days or weeks [11]. However, no indicative number of SABA episodes/week likely constituting an impending or ongoing asthma exacerbation is provided. Nor is it made clear how a patient’s baseline level of usage (from which the increase should be observed) should be determined. Clarity on these points is needed to support individualized asthma management.

In Phase 1 of our process, over half of the participants agreed that increased SABA usage indicated loss of asthma control. Our expert consensus reflects current SABA medication guidance and literature suggesting that patients using SABA on ≥2 days/week or for ≥2 SABA episodes/week have inadequately controlled asthma [16, 19]. Importantly, our Delphi consensus indicates that 2 to 3 (and, more strongly, ≥5) SABA episodes/week may be a more appropriate signal for an impending or ongoing exacerbation. It is important to note that the experts concurred that patients who exceeded their normal SABA use by 50 to 100% from their baseline level of usage are at a higher risk of an impending or ongoing exacerbation.

Weekly SABA use thresholds—both absolute and dynamic (changes from baseline behavior)—could help signal a patient’s increased risk of an impending or ongoing exacerbation, which requires prompt medical attention. Inclusion of such thresholds should be considered in future asthma guidelines.

While understanding weekly SABA use is important, SABA use history is also a useful indicator for clinicians to monitor reliever treatment. Most Phase 1 clinicians indicated that they obtain information about prior reliever use at every patient visit; almost all allergists and pulmonologists agreed with collecting SABA history in this way, whereas just over half of PCPs followed this practice. It was noted in Phase 3 that the need for prescription refills can present opportunity for discussions around a patient’s current level of asthma control. Improving guideline adherence in this setting, and so providing patients with access to best-practice management regardless of clinical setting and disease severity, is a key unmet need.

### The Unvarnished Truth: Accurately Monitoring SABA Use

A key question naturally arising from recognition of the value of individualized insights on SABA use regards the most effective way to accurately monitor actual use. Phase 3 participants acknowledged that patients need access to SABA, but questioned how this should be monitored. Most asthma guidelines consulted by Phase 1 participants cover general management [11, 16, 18–21]. While current asthma treatment guidelines emphasize monitoring SABA overuse, most lack detailed guidance on how to do this effectively and do not include specific recommendations for clinical action when a patient has already intensified maintenance therapy [11, 16, 18–21].

In Phase 1 of our process, patient history/recall was the most common way to assess SABA use (89% of clinicians). However, patient recall is subjective and can be inaccurate [22•]. Indeed, clinicians recognized the need to use other information, as they recognize that patient recall is only generally accurate (28% of clinicians) or is variable (49% of clinicians). Pharmacy refills were the second most common method to monitor SABA use (58% of clinicians), but refill data are inaccurate as they do not capture actual SABA use [48] and also do not lend themselves to acute intervention. Obtaining refill histories can also be difficult and time-consuming, particularly if multiple pharmacies need to be contacted. Moreover, availability of over-the-counter SABAs [3, 10••, 25] could be deleterious as accurate purchasing information would not be available.

The present expert panel favored using digital health tools where possible, as they provide objective, accurate, and reliable reliever usage and maintenance adherence data [22•, 48–50, 51•]. Such devices have the potential to support improvements in adherence and asthma control [51•, 52••], though other factors such as poor technique leading to unintentional nonadherence [53] and cost-related underuse [54] may also need to be addressed. In particular, as we aspire achievement of control/remission on therapy [55], the availability of objective insights on SABA use has potential to be of considerable clinical benefit. Phase 3 participants acknowledged the value of information about how SABA is used and its effect on patient’s level of asthma control.

While digital platforms for asthma management may not be needed by all patients (e.g., those with optimally controlled asthma), certain subpopulations with inadequately controlled asthma could benefit from their use; further research is needed to aid in guiding optimal use/patient selection. Asthma guidelines have yet to recommend digital tools in asthma, although this is a likely topic for future GINA updates [11].

Digital health tools would enable collection of SABA usage data at the granularity needed to enable clinicians to manage patients acutely in a more proactive and personalized way. Indeed, a recent study in adults with poorly controlled asthma, treated with an electronic multidose dry powder inhaler with integrated sensors, demonstrated that data collected by the digital inhaler could be used to develop a machine learning model capable of predicting impending exacerbations [56••].

Patients are increasingly embracing digital technology, many now having access to their own health information via apps and smart watches, for example [57]. Furthermore, they are starting to engage with these data and adjusting their own behavior as a result. There may come a time, in the not-too-distant future, where digital technology could be used to alert patients to changes in their asthma or patterns of inhaler use and enable them, and their physicians, to take the most appropriate action.

### Knowledge Is Power: Optimizing Clinical Decision-Making

The combination of objective data on patients’ real SABA use and expert guidance, such as that provided by our panel, has potential to substantially enhance clinical decision-making and so reduce exacerbations and improve patient outcomes.

We explored how clinicians currently respond to observed high SABA use. In Phase 1 of our process, the most frequently mentioned actions were medication change (76% of clinicians), inhaler technique training (60%), additional information gathering (60%), and asthma education refreshers (56%), whereas specialty referral was only mentioned by 22% of clinicians. The expert panel agreed that multiple interrelated clinical actions, including inhaler technique training, medication change, additional information gathering via phone or portal (specifically exploring triggers and comorbidities), and an asthma education refresher, should be considered in response to concerning patterns or levels of SABA use. Importantly, the experts emphasized that—while absolute thresholds might be used to identify patients at immediate risk of worsening—clinical asthma management should ideally be based on individual SABA use data (i.e., increase from baseline, usage patterns over time). This necessitates accurate determination of each patient’s typical usage. Information on patients’ day-to-day SABA usage patterns could contribute to more individualized treatment plans. However, this cannot be gleaned simply from claims data stating the number of refills per year. Some patients may have “spikes” of exacerbation-associated SABA use, interspersed between periods of no SABA use. Others with chronically poor asthma control may be consistently overusing SABA on almost a daily basis.

**Fig. 3:**
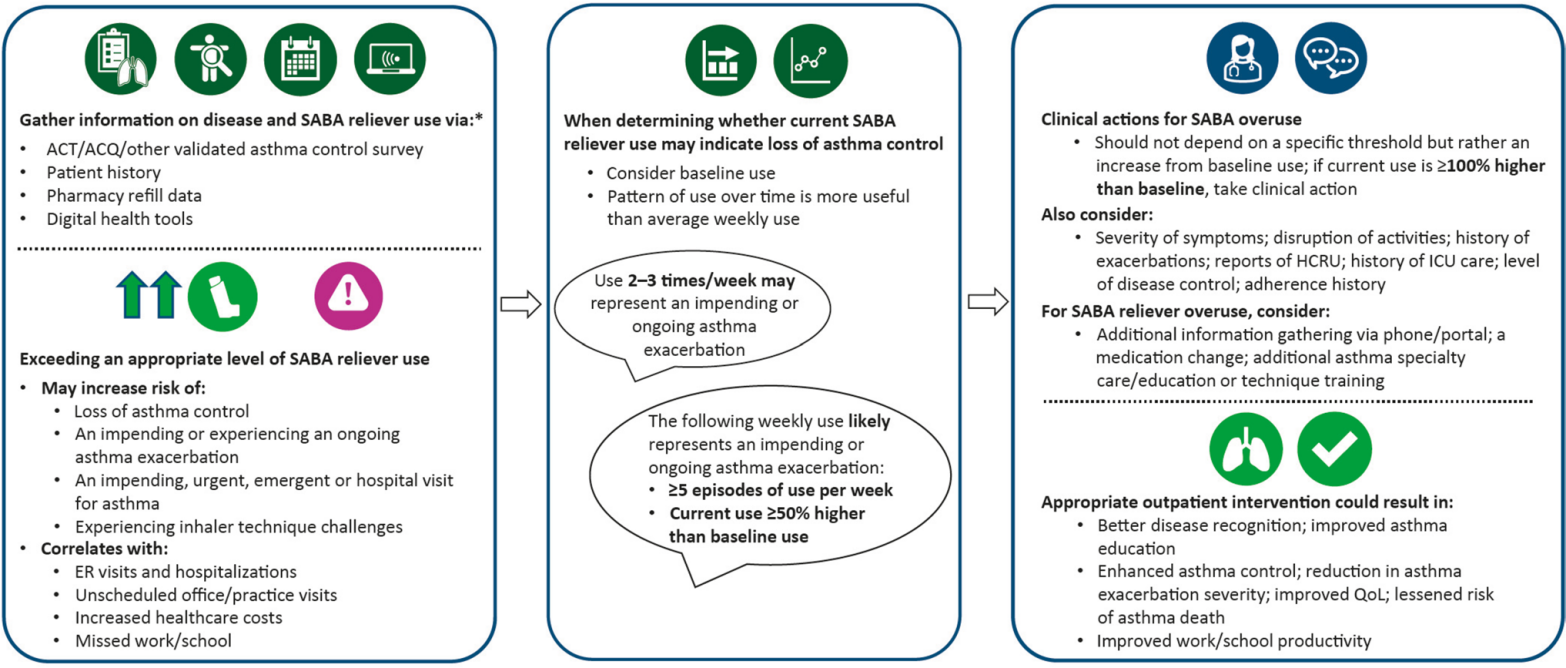
Putative framework for clinical application of consensus statements agreed in Phase 3. *ACT/ACQ/patient history are reliant on patient recall and may give inaccurate data. Pharmacy refill data provides a quantitative insight but does not provide information on usage pattern. Digital heath tools can provide an objective real-time dynamic insight into true SABA use. ACQ, Asthma Control Questionnaire; ACT, Asthma Control Test; HCRU, healthcare resource utilization; ICU, intensive care unit; QoL, quality of life; SABA, short-acting beta_2_ agonist

Thus, individual clinical judgment becomes essential, which is more-or-less reliant on the clinician’s experience and confidence in the specific scenario. The quality of objective information available to clinicians also strongly affects their ability to make rational decisions regarding treatment. The 70 consensus statements agreed upon in Phase 3 provide actionable thresholds for asthma clinical practice that could be adopted by clinicians to better monitor SABA usage and prescriptions on a patient-by-patient basis. Figure 3 provides a putative framework for clinical application of these thresholds. Together with more granular data from digital health tools, these consensus statements may support future updates to guidelines or clarify existing opinions around asthma management for SABA use.

### Limitations

The Delphi method is widely used in healthcare settings [58, 59] including asthma [60••, 61•]. However, this method has several well-recognized limitations. The present results are qualitative and should be considered as informative guidance only, which requires further objective evidence.

The Delphi process that we undertook was, by design, limited in its scope and sharply focused on SABA usage data and the information that these can provide about a patient’s disease status and treatment needs. Of note, exploration of individual patient factors underlying symptomatic disease was outside of the scope of this process.

Patterns of utilization of multiple inhalers by patients remain poorly understood. Although possessing several SABA inhalers may demonstrate overuse in some patients, others may prefer having several devices to ensure ready access at home, office, car, etc. Such usage should be understood to differentiate problematic versus cautious inhaler ownership.

## Conclusions

In this three-phase study, asthma experts recognized the risks of SABA overuse and recommended considering thresholds of SABA use for optimal clinical action based on understanding individual patient asthma clinical profiles. Therefore, gathering patient-specific insights, and improving the validity and reliability of SABA usage data, has the potential to substantially aid asthma management; this target is likely to be better achieved through the utilization of objective measurements of SABA use (e.g., digital inhalers or add-on monitoring devices).

Current guidelines express concerns over SABA overuse and no longer recommend SABA monotherapy. Increased use of digital health tools, enabling day-to-day monitoring and collection of more granular patient-level data, should support the advancement of future asthma guidelines; these could include specific recommendations regarding SABA use patterns, using expert-led thresholds for clinical action such as those described herein. As such, digital inhalers could potentially bridge the gap between guidelines and their implementation and support a more personalized approach to the treatment of asthma.

### Supplementary Information

Below is the link to the electronic supplementary material.

Supplementary file1 (DOCX 51 KB)

## Data Availability

The data sets used and/or analyzed for the study described in this manuscript are available upon reasonable request. Qualified researchers may request access to patient level data and related study documents including the study protocol and the statistical analysis plan. Patient level data will be de-identified and study documents will be redacted to protect the privacy of trial participants and to protect commercially confidential information. Please visit www.clinicalstudydatarequest.com to make your request.
