# Supplementary material for: Expert Consensus on SABA Use for Asthma Clinical Decision-Making: A Delphi Approach
Source: Curr Allergy Asthma Rep. 2023 Nov 22;23(11):621–34. doi: 10.1007/s11882-023-01111-z (PMC10716188; doi:10.1007/s11882-023-01111-z)
Supplement: Supplementary file 1 — Supplementary file1 (DOCX 51 KB) [file 11882_2023_1111_MOESM1_ESM.docx]

**Supplementary material for**

**Expert consensus on SABA use for asthma clinical decision-making: a Delphi approach**

Njira Lugogo, MD^a^, Maeve O’Connor, MD^b^, Maureen George, PhD RN^c^, Rajan Merchant, MD^d^, Greg Bensch, MD^e^, Jay Portnoy, MD^f^, John Oppenheimer, MD^g^, Mario Castro MD, MPH^h^

*^a^Division of Pulmonary & Critical Care Medicine, Department of Internal Medicine, University of Michigan, Ann Arbor, MI, USA;* *^b^Allergy Asthma and Immunology Relief, Charlotte, NC, USA;* *^c^Columbia University School of Nursing, New York, NY, USA;* *^d^Woodland Clinic Medical Group, Allergy Department, Dignity Health, Woodland, CA, USA;* *^e^Allergy Immunology and Asthma Medical Group, Stockton, CA, USA;* *^f^Section of Allergy, Asthma & Immunology, Children's Mercy Hospital, Kansas City, Missouri, University of Missouri-Kansas City School of Medicine, Kansas City, MO, USA;* *^g^Department of Internal Medicine, New Jersey Medical School, Newark, New Jersey; Pulmonary and Allergy Associates, Morristown, NJ, USA; ^h^Division of Pulmonary, Critical Care, and Sleep Medicine, Department of Internal Medicine, University of Kansas Medical Center, Kansas City, KS, USA*

**Corresponding author:** Njira Lugogo, MD, Division of Pulmonary & Critical Care Medicine, Department of Internal Medicine, University of Michigan, 300 North Ingalls St, Suite 2c40, Ann Arbor, MI 48109, USA. Email: [nlugogo@med.umich.edu](mailto:nlugogo@med.umich.edu)

**Appendix 1: Supplementary methods**

**Phases**

Phase 1 participants completed a 38-item online survey on practice patterns and beliefs around asthma SABA use, including six demographic questions (participant and medical practice) and 32 questions addressing core objectives. Anonymous survey responses were analyzed descriptively and, per Delphi recommendations, used to develop questions on asthma management and SABA use for Phase 2 discussions.

Phase 2 participants completed a pre-forum online survey (Appendix 2), comprising questions relating to asthma management and use of SABA reliever medication developed from the results from the Phase 1 survey, followed by an online forum discussion, via an internal proprietary, password-protected platform. As part of the forum, the participants answered several questions, reviewed and replied to each other’s comments via discussion boards, and responded to case studies for adjudication, to determine what clinical action might be taken in various scenarios of SABA reliever medication use in patients with asthma. Phase 1 results and Phase 2 insights were used to develop questions and statements about SABA reliever medication use and asthma management for Phase 3 discussions.

In Phase 3, participants completed a pre-meeting survey (Appendix 3), participated in a live video-conference discussion, and completed a post-meeting survey (Appendix 4). For the pre-meeting survey, to gauge the initial level of consensus with the statements developed to address the initial domains of the project, panelists rated each statement using a five-point Likert scale: 1) strongly disagree; 2) disagree; 3) neither agree nor disagree; 4) agree; 5) strongly agree.^1^ To determine which statements reached consensus, median score and interquartile range were calculated for each. Consensus to reject a statement occurred if the upper quartile was ≤2, and consensus to accept a statement occurred if the lower quartile was ≥4. Statements meeting consensus during the pre-meeting survey were included in the final list without further discussion. Statements lacking consensus required further review and refinement and were discussed in a video conference. The video conference included open discussion and online real-time polling via the Zoom meeting platform. For some statements, additional discussion led to a change in opinion among participants and a re-vote was taken using the original wording. In other cases, participants made suggestions for revisions that allowed the group to reach a consensus. The participants could also decide to drop some statements from discussion and not conduct a re-vote. In these cases, the decision to drop was due to lack of opinion, lack of importance, or lack of evidence to support opinion. Statements not sufficiently discussed during the video conference were presented for a final consensus vote through the post-meeting survey.

**Appendix 2. Phase 2 pre-forum survey.**

1. What level of risk for negative outcomes (e.g., exacerbation, emergency visit, hospitalization) might exceeding the amount/threshold of SABA use you feel is appropriate pose to the patient? (Very high risk, high risk, some risk, little risk, no risk)

2. With an impending exacerbation as supported by known SABA use data, how far in advance could an appropriate outpatient treatment prove of benefit and prevent that exacerbation from occurring? (Minutes, Hours, Days, Weeks)

3. How likely do you think an intervention might be to provide benefit within the timeframe you chose above? (Very likely to provide benefit, somewhat likely to provide benefit, unsure, somewhat unlikely to provide benefit, very unlikely to provide benefit)

4. Which of the following might be affected in the future for the patient if a “SABA use-connected” exacerbation is lessened in severity or prevented altogether? (Health care costs, hospitalization potential, unscheduled visit potential, patient activity, quality of life, disease progression, other)

5. When considering SABA rescue medication use, what literature, expert reports and/or guidelines help inform your clinical decisions? NIH/NAEPP, GINA, ERS, ATS, Baylor University “Rules of Two”, None, Other)

6. Do you feel the literature, expert reports and/or guidelines you chose above provide clear recommendations of effective and safe amounts/thresholds of SABA rescue medication use? (Yes, no, unsure)

7. Do you feel those literature, expert reports and/or guidelines provide clear recommendations on what clinical action to take and when in response to SABA rescue medication overuse? (Yes, no, unsure)

**Appendix 3: Phase 3 pre-meeting survey.**

Participants rated the following list of statements using a 5-point Likert scale, with the response options: 1) Strongly disagree; 2) Disagree; 3) Neither agree nor disagree; 4) Agree; 5) Strongly agree.

1. The Asthma Control Test (ACT), Asthma Control Questionnaire (ACQ) or another validated asthma control survey should be used as part of routine assessment of patients with asthma. (Please select only one)

2. The amount of a patient’s rescue medication use should be part of their asthma medical history evaluations. (Please select only one)

3. For patients who have been prescribed SABA rescue medication (e.g., albuterol) for asthma, information about previous rescue medication use in the prior weeks or months should be obtained at every visit. (Please select only one)

4. Patient history should be used to assess SABA rescue medication use for patients with asthma. (Please select only one)

5. Validated questionnaires (e.g. ACQ) should be used to assess SABA rescue medication use for patients with asthma. (Please select only one)

6. Pharmacy refill data should be used to assess SABA rescue medication use for patients with asthma. (Please select only one)

7. Digital health tools should be used to assess SABA rescue medication use for patients with asthma. (Please select only one).

8. A patient’s asthma rescue medication use should factor into clinical decision-making for asthma management. (Please select only one)

9. A better understanding of SABA rescue medication overuse should play a role in shared decision-making between patients with asthma and healthcare professionals in the United States. (Please select only one)

10. Patient SABA use history is generally accurate, and no other assessment tools should be used to understand the patient’s SABA use since their previous visit. (Please select only one)

11. Patient SABA use history is generally accurate, but other information (e.g. validated questionnaires, refill data, digital recorders) should be used to obtain accurate information on the patient’s SABA use since their previous visit. (Please select only one)

12. The accuracy of patient SABA use history is variable, and its use should depend on the patient. (Please select only one)

13. The accuracy of patient SABA use history is generally inaccurate and should not be used to determine the patient’s SABA use since their previous visit. (Please select only one)

14. SABA rescue use frequency as gathered in patient history should play a significant role in the assessment of a patient's asthma disease severity. (Please select only one)

15. SABA rescue use frequency should play a substantial role in the assessment of a patient’s asthma disease control. (Please select only one)

Patients with asthma who are prescribed a SABA inhaler for as needed rescue should receive: (Please select an option for each row)

16. No refills

17. No more than 1 refill

18. No more than 2 refills

19. No more than 3 refills

20. 4 or more refills (prn is fine)

If a patient exceeds the level of SABA rescue medication use you feel is appropriate, they may be at risk of: (Please select an option for each row)

21. Loss of asthma control

22. An impending or experiencing an ongoing asthma exacerbation

23. An impending, urgent, emergent or hospital visit for asthma

24. Inappropriate SABA use

25. Experiencing inhaler technique challenges

26. Nothing. There would be no risk of negative outcomes

If a patient exceeds the level of SABA rescue medication use you feel is appropriate, they are likely to be at risk of: (Please select an option for each row)

27. Loss of asthma control

28. An impending or experiencing an ongoing asthma exacerbation

29. An impending, urgent, emergent or hospital visit for asthma

30. Inappropriate SABA use

31. Experiencing inhaler technique challenges

32. If a patient exceeds the level of SABA rescue medication use you feel is appropriate, it is not likely that they are at risk of any negative outcome

The following amount of weekly SABA rescue medication use likely represents an impending or ongoing asthma exacerbation: (please select an option for each row)

33. 1 or more episodes of SABA rescue medication use per week

34. 2 or more episodes of SABA rescue medication use per week

35. 3 or more episodes of SABA rescue medication use per week

36. 5 or more episodes of SABA rescue medication use per week

37. 7 or more episodes of SABA rescue medication use per week

38. 10 or more episodes of SABA rescue medication use per week

39. 15 or more episodes of SABA rescue medication use per week

40. 20 or more episodes of SABA rescue medication use per week

41. 25 or more episodes of SABA rescue medication use per week

42. The patient’s baseline SABA rescue medication use should be considered when determining whether the current weekly use may indicate an impending or ongoing exacerbation. (Please select only one)

43. If the patient’s current SABA rescue medication use is 50% higher than their baseline use, this likely represents an impending or ongoing exacerbation. (Please select only one)

44. If the patient’s current SABA rescue medication use is 100% higher than their baseline use or more, this likely represents an impending or ongoing exacerbation. (Please select only one)

45. The pattern of SABA use over time should play a role in determining whether SABA rescue medication use might represent an exacerbation. (Please select only one)

46. The patient’s SABA rescue medication use pattern over time is more useful than an average of weekly SABA rescue medication use for determining whether a patient may be experiencing an impending or ongoing exacerbation. (Please select only one)

47. Valid historical data about night-time SABA asthma rescue medication use should factor into a clinician’s level of concern about asthma exacerbations. (Please select only one)

48. If it were possible, knowing of an impending exacerbation hours in advance would allow for an outpatient medication intervention that could prevent the exacerbation. (Please select only one)

49. If it were possible, knowing of an impending exacerbation days in advance would allow for an outpatient medication intervention that could prevent the exacerbation. (Please select only one)

50. If it were possible, knowing of an impending exacerbation weeks in advance would allow for an outpatient medication intervention that could prevent the exacerbation. (Please select only one)

51. The patient’s baseline SABA rescue medication use should be considered when determining whether the current weekly use may indicate a loss of asthma control. (Please select only one)

52. If the patient’s current SABA rescue medication use is 50% higher than their baseline use, this likely represents a loss of asthma control. (Please select only one)

53. If the patient’s current SABA rescue medication use is 100% higher than their baseline use or more, this likely represents a loss of asthma control. (Please select only one)

54. The pattern of SABA use over time should play a role in determining whether SABA rescue medication use might represent a loss of asthma control. (Please select only one)

55. The patient’s SABA rescue medication use pattern over time is more useful than an average of weekly SABA rescue medication use for determining loss of asthma control. (Please select only one)

56. SABA rescue medication overuse could indicate suboptimal effectiveness of a patient’s asthma maintenance therapy. (Please select only one)

There is a correlation between overuse of SABA rescue medications and: (Please select an option for each row)

57. Practice visits

58. ER visits

59. Hospitalizations

60. Unscheduled office/practice visits

61. Increased healthcare costs

62. Missed work/school

63. Additional information gathering via phone/portal should be considered if a patient is overusing their SABA rescue medication. (Please select only one)

64. A medication change should be considered if a patient is overusing their SABA rescue medication. (Please select only one)

65. An asthma education refresher should be considered if a patient is overusing their SABA rescue medication. (Please select only one)

66. Inhaler technique training should be considered if a patient is overusing their SABA rescue medication. (Please select only one)

67. Clinical actions in response to a patient overusing their SABA rescue medication should depend on a specific threshold amount of weekly SABA rescue medication overuse. (Please select only one)

68. The patient’s baseline SABA rescue medication use should be considered when determining whether the current weekly use should warrant additional clinical action. (Please select only one)

69. If the current SABA rescue medication use is 50% higher than the patient’s baseline, this should warrant additional clinical action. (Please select only one)

70. If the current SABA rescue medication use is 100% higher or more than the patient’s baseline, this should warrant additional clinical action. (Please select only one)

The following should influence the decision about how to respond to a patient's SABA rescue medication overuse: (Please select an option for each row)

71. Severity of symptoms

72. Disruption of activities

73. History of exacerbations

74. Reports of healthcare utilization (e.g., urgent care, ER, hospitalization)

75. History of ICU care

76. Level of disease control

77. Adherence history

The following should be considered for patients identified as overusing their SABA rescue medication overuse: (Please select an option for each row)

78. Additional asthma specialty care

79. Additional inhaler training

80. Additional asthma education

81. Additional breathing exercise training

82. Social services care

An appropriate outpatient asthma therapeutic intervention for a patient with asthma demonstrating excessive SABA rescue medication use could result in: (Please select an option for each row)

83. Better disease recognition

84. Improved asthma education

85. Enhanced asthma control

86. Reduction in asthma exacerbation severity

87. Improved quality of life

88. Lessened risk of asthma death

89. Improved work/school productivity

90. Socioeconomic status (SES) influences a patient’s need for SABA rescue medication use. (Please select only one)

91. Socioeconomic status should influence the number of SABA rescue medication refills provided to a patient. (Please select only one)

92. Patients of lower SES should receive more SABA rescue medication refills than a patient with higher SES. (Please select only one)

93. Patients of lower SES should receive less SABA rescue medication refills than a patient with higher SES. (Please select only one)

94. Current asthma expert report/guidelines provide clear recommendations regarding effective and safe amounts of SABA use as rescue medication. (Please select only one)

95. Current asthma report/guidelines provide clear recommendations on when to take clinical action in response to excessive SABA rescue medication use. (Please select only one)

96. Current asthma report/guidelines provide clear recommendations on what clinical action to take in response to SABA rescue medication overuse. (Please select only one)

97. Current asthma report/guideline provide clear recommendations regarding effective and safe amounts of SABA for proactive use (e.g., prior to exercise). (Please select only one)

**Appendix 4: Phase 3 post-meeting survey.**

Participants rated the following list of statements using a 5-point Likert scale, with the response options: 1) Strongly disagree; 2) Disagree; 3) Neither agree nor disagree; 4) Agree; 5) Strongly agree.

1. Current asthma expert report/guidelines provide clear recommendations regarding effective and safe amounts of SABA use as rescue medication. (Please select only one)

2. Current asthma report/guidelines provide clear recommendations on when to take clinical action in response to excessive SABA rescue medication use. (Please select only one)

3. Current asthma report/guidelines provide clear recommendations on what clinical action to take in response to SABA rescue medication overuse. (Please select only one)

4. Current asthma report/guideline provide clear recommendations regarding effective and safe amounts of SABA for proactive use (i.e., prior to exercise). (Please select only one)

5. Socioeconomic status should influence the number of SABA rescue medication refills provided to a patient. (Please select only one)

6. The following should be considered for patients identified as overusing their SABA rescue medication: (Please select an option for each row)

a. Additional breathing exercise training

b. Social services care

7. There is a correlation between overuse of SABA rescue medications and practice visits. (Please select only one)

8. If the patient’s current SABA rescue medication use is 50% higher than their baseline use, this likely represents a loss of asthma control. (Please select only one)

9. If the patient’s current SABA rescue medication use is 100% higher than their baseline use or more, this likely represents a loss of asthma control. (Please select only one)

**Appendix 5: Phase 1 online survey results.**

| Response | Primary care physician n (%) (n = 50) | Allergist n (%)  (n = 35) | Pulmonologist n (%)  (n = 15) |
| --- | --- | --- | --- |
| Does your practice use the Asthma Control Test, Asthma Control Questionnaire or another validated asthma control survey as part of your routine assessment of patients with asthma? | | | |
| Yes | 22 (44) | 29 (83) | 9 (60) |
| No | 28 (56) | 6 (17) | 6 (40) |
| To what extent does your practice find it important to understand the amount of a patient’s rescue medication use during asthma medical history evaluations? | | | |
| A very large extent | 23 (46) | 21 (60) | 7 (47) |
| A large extent | 17 (34) | 12 (34) | 6 (40) |
| A moderate extent | 8 (16) | 2 (6) | 2 (13) |
| A small extent | 2 (4) | 0 (0) | 0 (0) |
| A very small extent/not at all | 0 (0) | 0 (0) | 0 (0) |
| To what extent does the amount of a patient’s asthma rescue medication use factor into your clinical decision-making for asthma management? | | | |
| A very large extent | 22 (44) | 22 (63) | 7 (47) |
| A large extent | 19 (38) | 11 (31) | 5 (33) |
| A moderate extent | 8 (16) | 2 (6) | 3 (20) |
| A small extent | 1 (2) | 0 (0) | 0 (0) |
| A very small extent/not at all | 0 (0) | 0 (0) | 0 (0) |
| For your patients who have been prescribed SABA rescue medication (e.g., albuterol) for asthma, how frequently do you obtain information about previous rescue medication use in the prior weeks or months? | | | |
| Every visit | 28 (56) | 32 (91) | 13 (87) |
| Most visits | 17 (34) | 3 (9) | 2 (13) |
| Occasionally, depending on visit factors | 5 (10) | 0 (0) | 0 (0) |
| I usually do not ask this question | 0 (0) | 0 (0) | 0 (0) |
| If an asthma patient is prescribed a SABA inhaler for as needed rescue, how often does your practice provide more than one SABA canister per prescription? | | | |
| Never | 4 (8) | 9 (26) | 2 (13) |
| <25% of the time | 21 (42) | 13 (37) | 5 (33) |
| 25–50% of the time | 15 (30) | 6 (17) | 4 (27) |
| 51–75% of the time | 8 (16) | 1 (3) | 4 (27) |
| 76–100% of the time | 2 (4) | 6 (17) | 0 (0) |
| If a patient with asthma is prescribed a SABA inhaler for as needed rescue, how many refills does your practice typically provide? | | | |
| 0 refills | 2 (4) | 10 (29) | 0 (0) |
| 1 refill | 5 (10) | 9 (26) | 3 (20) |
| 2 refills | 15 (30) | 9 (26) | 4 (27) |
| 3 refills | 18 (36) | 5 (14) | 8 (53) |
| 4 refills | 0 (0) | 1 (3) | 0 (0) |
| 5 refills | 3 (6) | 0 (0) | 0 (0) |
| 6 or more refills (= ”refill prn”) | 7 (14) | 1 (3) | 0 (0) |
| What technique does your practice typically use to assess SABA rescue medication use for patients with asthma? | | | |
| Digital health information | 9 (18) | 4 (11) | 3 (20) |
| Patient history/patient recall | 42 (84) | 33 (94) | 14 (93) |
| Pharmacy refills data | 31 (62) | 18 (51) | 9 (60) |
| Validated questionnaires | 8 (16) | 15 (43) | 4 (27) |
| Other | 0 (0) | 0 (0) | 0 (0) |
| None of the above | 1 (2) | 0 (0) | 0 (0) |
| Which of the following statements best reflects your opinion on the accuracy of asthma patient history/patient recall with respect to SABA rescue medication use since the patient’s previous visit? | | | |
| Patient SABA use history is generally accurate; I need no other assessment tool | 11 (22) | 1 (3) | 2 (13) |
| Patient SABA use history is generally accurate, but I use other  information too | 15 (30) | 11 (31) | 2 (13) |
| The accuracy of patient SABA use history is variable, I use this  information depending on the patient | 22 (44) | 19 (54) | 8 (53) |
| Patient SABA use history is generally inaccurate, but I use it with other information | 2 (4) | 4 (11) | 3 (20) |
| Patient SABA use history is generally inaccurate; I prefer to not ask about SABA use and rely more on other assessment(s) | 0 (0) | 0 (0) | 0 (0) |
| To what extent do you rely on SABA rescue use frequency to inform your assessment about asthma disease severity? | | | |
| A very large extent | 13 (26) | 13 (37) | 5 (33) |
| A large extent | 22 (44) | 12 (34) | 5 (33) |
| A moderate extent | 13 (26) | 9 (26) | 5 (33) |
| A small extent | 2 (4) | 1 (3) | 0 (0) |
| A very small extent/not at all | 0 (0) | 0 (0) | 0 (0) |
| To what extent do you rely on SABA rescue use frequency to inform your assessment about asthma disease control? | | | |
| A very large extent | 14 (28) | 12 (34) | 6 (40) |
| A large extent | 23 (46) | 16 (46) | 6 (40) |
| A moderate extent | 10 (20) | 7 (20) | 3 (20) |
| A small extent | 3 (6) | 0 (0) | 0 (0) |
| A very small extent/not at all | 0 (0) | 0 (0) | 0 (0) |
| If an asthma patient exceeds the level of SABA rescue medication use you feel is appropriate, what risk might this represent? | | | |
| Impending or ongoing asthma exacerbation | 40 (80) | 27 (77) | 13 (87) |
| Impending urgent, emergent, or hospital visit for asthma | 28 (56) | 23 (66) | 12 (80) |
| Inappropriate use | 29 (58) | 26 (74) | 11 (73) |
| Inhaler technique challenges | 26 (52) | 25 (71) | 10 (67) |
| Loss of asthma control | 34 (68) | 33 (94) | 12 (80) |
| Other | 1 (2) | 0 (0) | 0 (0) |
| None | 0 (0) | 0 (0) | 0 (0) |
| If an asthma patient exceeds the level of SABA rescue medication use you feel is appropriate, what risk does this most often represent in your practice? | | | |
| Impending or ongoing asthma exacerbation | 18 (36) | 9 (26) | 2 (13) |
| Impending urgent, emergent, or hospital visit for asthma | 6 (12) | 1 (3) | 4 (27) |
| Inappropriate use | 2 (4) | 2 (6) | 2 (13) |
| Inhaler technique challenges | 0 (0) | 1 (3) | 0 (0) |
| Loss of asthma control | 24 (48) | 22 (63) | 7 (47) |
| Other | 0 (0) | 0 (0) | 0 (0) |
| None | 0 (0) | 0 (0) | 0 (0) |
| In your practice, what is the lowest amount of weekly SABA rescue medication need (measured by the number of episodes requiring SABA at any dose) that you believe possibly represents an impending or ongoing asthma exacerbation? | | | |
| None; the amount of asthma rescue medication use does not typically signal an impending or ongoing exacerbation | 1 (2) | 0 (0) | 1 (7) |
| ≥1 episodes per week | 9 (18) | 1 (3) | 2 (13) |
| ≥2 episodes per week | 11 (22) | 14 (40) | 7 (47) |
| ≥3 episodes per week | 16 (32) | 12 (34) | 4 (27) |
| ≥5 episodes per week | 8 (16) | 7 (20) | 1 (7) |
| ≥7 episodes per week | 4 (8) | 0 (0) | 0 (0) |
| ≥10 episodes per week | 0 (0) | 0 (0) | 0 (0) |
| ≥15 episodes per week | 1 (2) | 1 (3) | 0 (0) |
| ≥20 episodes per week | 0 (0) | 0 (0) | 0 (0) |
| ≥25 episodes per week | 0 (0) | 0 (0) | 0 (0) |
| In your practice, what is the lowest amount of weekly SABA rescue medication need (measured by the number of episodes requiring SABA at any dose) that you believe likely represents an impending or ongoing asthma exacerbation? | | | |
| None; the amount of asthma rescue medication use does not typically signal an impending or ongoing exacerbation | 1 (2) | 0 (0) | 1 (7) |
| ≥1 episodes per week | 5 (10) | 1 (3) | 2 (13) |
| ≥2 episodes per week | 10 (20) | 6 (17) | 3 (20) |
| ≥3 episodes per week | 12 (24) | 12 (34) | 2 (13) |
| ≥5 episodes per week | 9 (18) | 11 (31) | 5 (33) |
| ≥7 episodes per week | 7 (14) | 3 (9) | 1 (7) |
| ≥10 episodes per week | 5 (10) | 1 (3) | 1 (7) |
| ≥15 episodes per week | 1 (2) | 0 (0) | 0 (0) |
| ≥20 episodes per week | 0 (0) | 1 (3) | 0 (0) |
| ≥25 episodes per week | 0 (0) | 0 (0) | 0 (0) |
| In your practice, what is the lowest amount of SABA rescue medication needed by a patient (measured by the number of episodes requiring SABA at any dose) that would typically prompt telephone follow-up or any other information gathering by your practice? | | | |
| None; the amount of SABA asthma rescue medication use does not matter in clinical decision-making | 1 (2) | 0 (0) | 0 (0) |
| ≥1 episodes per week | 2 (4) | 0 (0) | 2 (13) |
| ≥2 episodes per week | 14 (28) | 10 (29) | 2 (13) |
| ≥3 episodes per week | 12 (24) | 12 (34) | 6 (40) |
| ≥5 episodes per week | 9 (18) | 7 (20) | 3 (20) |
| ≥7 episodes per week | 2 (4) | 3 (9) | 2 (13) |
| ≥10 episodes per week | 8 (16) | 2 (6) | 0 (0) |
| ≥15 episodes per week | 1 (2) | 1 (3) | 0 (0) |
| ≥20 episodes per week | 1 (2) | 0 (0) | 0 (0) |
| ≥25 episodes per week | 0 (0) | 0 (0) | 0 (0) |
| Which of the following clinical actions might be considered if you feel a patient is using their SABA rescue medication more than what you consider an appropriate threshold amount? | | | |
| Additional information gathering via phone/portal | 21 (42) | 26 (74) | 9 (60) |
| Asthma education refresher | 22 (44) | 20 (57) | 6 (40) |
| Inhaler technique training | 27 (54) | 25 (71) | 8 (53) |
| Medication change | 42 (84) | 24 (69) | 11 (73) |
| Specialty referral | 15 (30) | 2 (6) | 2 (13) |
| Other | 2 (4) | 0 (0) | 0 (0) |
| Which of the following clinical actions should be considered if you feel a patient is overusing their SABA rescue medication? | | | |
| Additional information gathering via phone/portal | 26 (52) | 26 (74) | 8 (53) |
| Asthma education refresher | 32 (64) | 17 (49) | 7 (47) |
| Inhaler technique training | 28 (56) | 25 (71) | 7 (47) |
| Medication change | 39 (78) | 25 (71) | 12 (80) |
| Specialty referral | 16 (32) | 2 (6) | 4 (27) |
| Other | 1 (2) | 0 (0) | 0 (0) |
| To what extent does your choice of clinical action in response to a patient overusing their SABA rescue medication depend on a specific threshold amount of that SABA rescue medication overuse? | | | |
| A very large extent | 4 (8) | 6 (17) | 2 (13) |
| A large extent | 23 (46) | 15 (43) | 7 (47) |
| A moderate extent | 18 (36) | 12 (34) | 6 (40) |
| A small extent | 4 (8) | 2 (6) | 0 (0) |
| A very small extent/not at all | 1 (2) | 0 (0) | 0 (0) |
| In practice, what other factors may influence your decision about how to respond to a patient’s SABA rescue medication overuse? | | | |
| Disease control | 6 (12) | 8 (23) | 3 (20) |
| Disruption of activities | 24 (48) | 18 (51) | 8 (53) |
| History of exacerbations | 35 (70) | 25 (71) | 12 (80) |
| History of ICU | 4 (8) | 2 (6) | 0 (0) |
| Reports of HCRU (e.g. urgent care, ER visit, hospitalization) | 33 (66) | 20 (57) | 6 (40) |
| Severity of symptoms | 33 (66) | 29 (83) | 11 (73) |
| If you would choose to refer a patient for additional care after identifying overuse of SABA rescue medication, what type(s) of (internal or external) referral might be considered most frequently in your practice? | | | |
| Additional asthma specialty care | 30 (60) | 12 (34) | 8 (53) |
| Asthma education | 31 (62) | 28 (80) | 10 (67) |
| Breathing exercises | 10 (20) | 3 (9) | 3 (20) |
| Inhaler training | 27 (54) | 28 (80) | 12 (80) |
| Social services | 1 (2) | 4 (11) | 0 (0) |
| Other | 0 (0) | 0 (0) | 0 (0) |
| I would not choose to refer the patient for additional care | 3 (6) | 5 (14) | 2 (13) |
| How might an appropriate outpatient asthma therapeutic intervention provided by your practice benefit a patient with asthma demonstrating excessive SABA rescue medication use? | | | |
| Better disease recognition | 16 (32) | 17 (49) | 4 (27) |
| Enhanced asthma control | 31 (62) | 29 (83) | 11 (73) |
| Exacerbation prevention | 37 (74) | 26 (74) | 9 (60) |
| Improved asthma education | 27 (54) | 26 (74) | 10 (67) |
| Improved productivity | 19 (38) | 17 (49) | 7 (47) |
| Improved QoL | 38 (76) | 23 (66) | 10 (67) |
| Lessened risk of asthma death | 21 (42) | 18 (51) | 6 (40) |
| Lessened risk of asthma hospitalization | 28 (56) | 20 (57) | 7 (47) |
| Lessened risk of asthma urgent care or ER visit | 26 (52) | 25 (71) | 8 (53) |
| Reduction in asthma exacerbation severity | 31 (62) | 20 (57) | 7 (47) |
| Other | 1 (2) | 0 (0) | 0 (0) |
| None | 0 (0) | 0 (0) | 0 (0) |
| To what extent do you believe SABA rescue medication overuse might indicate suboptimal effectiveness of an asthma patient’s maintenance therapy? | | | |
| A very large extent | 11 (22) | 10 (29) | 3 (20) |
| A large extent | 26 (52) | 17 (49) | 10 (67) |
| A moderate extent | 12 (24) | 8 (23) | 2 (13) |
| A small extent | 1 (2) | 0 (0) | 0 (0) |
| A very small extent/not at all | 0 (0) | 0 (0) | 0 (0) |
| To what extent do you believe SABA rescue medication overuse might indicate suboptimal inhaler technique in a patient who uses a SABA inhaler? | | | |
| A very large extent | 3 (6) | 6 (17) | 2 (13) |
| A large extent | 22 (44) | 10 (29) | 4 (27) |
| A moderate extent | 13 (26) | 12 (34) | 8 (53) |
| A small extent | 12 (24) | 7 (20) | 1 (7) |
| A very small extent/not at all | 0 (0) | 0 (0) | 0 (0) |
| To what extent do you believe an asthma patient’s SES influences their need for SABA rescue medication use? | | | |
| A very large extent | 7 (14) | 5 (14) | 0 (0) |
| A large extent | 12 (24) | 9 (26) | 6 (40) |
| A moderate extent | 21 (42) | 11 (31) | 8 (53) |
| A small extent | 9 (18) | 8 (23) | 1 (7) |
| A very small extent/not at all | 1 (2) | 2 (6) | 0 (0) |
| Which of the following statements best reflects your thoughts about an increased need for rescue medication among those of lower SES? | | | |
| I am concerned and very likely to increase SABA prescription refills for those of lower SES | 4 (8) | 2 (6) | 2 (13) |
| I am concerned and likely to increase SABA prescription refills for those of lower SES | 14 (28) | 7 (20) | 4 (27) |
| I am concerned but the SES does not influence my SABA prescribing habits | 26 (52) | 18 (51) | 7 (47) |
| I am concerned and likely to be more restrictive of SABA prescription refills for those of lower SES | 6 (12) | 8 (23) | 1 (7) |
| I am concerned and very likely to be more restrictive of SABA prescriptions refills for those of lower SES | 0 (0) | 0 (0) | 1 (7) |
| In which of the following, do you see a correlation between overuse of SABA rescue medications and rates of healthcare utilization? | | | |
| ER visits | 41 (82) | 27 (77) | 14 (93) |
| Hospitalizations | 30 (60) | 22 (63) | 13 (87) |
| Increased healthcare costs | 22 (44) | 16 (46) | 6 (40) |
| Missed work/school | 24 (48) | 22 (63) | 8 (53) |
| Practice visits in general | 16 (32) | 15 (43) | 6 (40) |
| Unscheduled visits | 23 (46) | 25 (71) | 10 (67) |
| Other | 1 (2) | 0 (0) | 0 (0) |
| If it were possible, how far in advance of an exacerbation might an appropriate outpatient medical intervention provided by your practice prevent that exacerbation? | | | |
| Minutes | 5 (10) | 1 (3) | 1 (7) |
| Hours | 17 (34) | 5 (14) | 4 (27) |
| Days | 26 (52) | 28 (80) | 7 (47) |
| Weeks | 2 (4) | 1 (3) | 3 (20) |
| To what extent does valid historical data about night-time need for SABA asthma rescue medication influence your asthma exacerbation concern? | | | |
| A very large extent | 9 (18) | 11 (31) | 2 (13) |
| A large extent | 22 (44) | 13 (37) | 6 (40) |
| A moderate extent | 16 (32) | 10 (29) | 7 (47) |
| A small extent | 3 (6) | 1 (3) | 0 (0) |
| A very small extent/not at all | 0 (0) | 0 (0) | 0 (0) |
| To what extent do you think a better understanding of SABA rescue medication overuse might improve communication/shared decision-making between patients with asthma and healthcare professionals in the U.S.? | | | |
| A very large extent | 7 (14) | 10 (29) | 2 (13) |
| A large extent | 22 (44) | 12 (34) | 10 (67) |
| A moderate extent | 17 (34) | 12 (34) | 3 (20) |
| A small extent | 4 (8) | 1 (3) | 0 (0) |
| A very small extent/not at all | 0 (0) | 0 (0) | 0 (0) |
| In your asthma management practice, which (if any) of the following asthma guideline/expert report recommendations do you routinely use for SABA rescue medication guidance? (Choose one most frequently used.) | | | |
| ATS | 7 (14) | 2 (6) | 2 (13) |
| Baylor University Rules of Two^®^ | 1 (2) | 5 (14) | 0 (0) |
| ERS | 2 (4) | 0 (0) | 0 (0) |
| GINA | 5 (10) | 9 (26) | 9 (60) |
| NIH/NAEPP | 13 (26) | 19 (54) | 4 (27) |
| Other | 1 (2) | 0 (0) | 0 (0) |
| None | 21 (42) | 0 (0) | 0 (0) |
| I feel that the asthma expert report/guideline I use provides clear recommendations regarding effective and safe amounts of SABA use as rescue medication. | | | |
| Strongly agree | 4 (8) | 9 (26) | 3 (20) |
| Agree | 30 (60) | 24 (69) | 10 (67) |
| Undecided | 13 (26) | 2 (6) | 2 (13) |
| Disagree | 2 (4) | 0 (0) | 0 (0) |
| Strongly disagree | 1 (2) | 0 (0) | 0 (0) |
| I feel that the asthma report/guideline I use provides clear recommendations on when to take clinical action in response to excessive SABA rescue medication use. | | | |
| Strongly agree | 8 (16) | 8 (23) | 3 (20) |
| Agree | 29 (58) | 24 (69) | 9 (60) |
| Undecided | 11 (22) | 3 (9) | 3 (20) |
| Disagree | 2 (4) | 0 (0) | 0 (0) |
| Strongly disagree | 0 (0) | 0 (0) | 0 (0) |
| I feel that the asthma report/guideline I use provides clear recommendations on what clinical action to take in response to SABA rescue medication overuse. | | | |
| Strongly agree | 11 (22) | 6 (17) | 3 (20) |
| Agree | 27 (54) | 24 (69) | 12 (80) |
| Undecided | 9 (18) | 5 (14) | 0 (0) |
| Disagree | 3 (6) | 0 (0) | 0 (0) |
| Strongly disagree | 0 (0) | 0 (0) | 0 (0) |
| I feel that the asthma report/guideline I use provides clear recommendations regarding effective and safe amounts of SABA for proactive use (e.g., prior to exercise). | | | |
| Strongly agree | 7 (14) | 8 (23) | 2 (13) |
| Agree | 32 (64) | 22 (63) | 10 (67) |
| Undecided | 9 (18) | 3 (9) | 3 (20) |
| Disagree | 2 (4) | 2 (6) | 0 (0) |
| Strongly disagree | 0 (0) | 0 (0) | 0 (0) |

ATS, American Thoracic Society; ER, emergency room; ERS, European Respiratory Society;
GINA, Global Initiative for Asthma; HCRU, healthcare resource utilization; ICU, intensive care unit; NIH/NAEPP, National Institutes of Health/National Asthma Education and Prevention Program;
QoL, quality of life; SABA, short-acting beta_2_-agonist; SES, socioeconomic status.

**References**

1. 5-Point Likert Scale. New York, NY: Springer New York; 2010. Accessed January 30, 2023 https://doi.org/10.1007/978-0-387-78665-0_6363.
